# Non-pharmacological interventions for bone health after stroke: A systematic review

**DOI:** 10.1371/journal.pone.0263935

**Published:** 2022-02-23

**Authors:** Hakimah Sallehuddin, Terence Ong, Salmiah Md. Said, Noor Azleen Ahmad Tarmizi, Siew Ping Loh, Wan Chieh Lim, Reena Nadarajah, Hong Tak Lim, Nurul Huda Mohd Zambri, Yun Ying Ho, Sazlina Shariff Ghazali

**Affiliations:** 1 Geriatric Medicine Unit, Department of Medicine, Faculty of Medicine and Health Sciences, Universiti Putra Malaysia, Selangor, Malaysia; 2 Malaysian Research Institute on Ageing (MyAgeingTM), Universiti Putra Malaysia, Selangor, Malaysia; 3 Division of Geriatric Medicine, Department of Medicine, Faculty of Medicine, University of Malaya, Kuala Lumpur, Malaysia; 4 Department of Community Health, Faculty of Medicine and Health Sciences, Universiti Putra Malaysia, Selangor, Malaysia; 5 Department of Medicine, Faculty of Medicine, Universiti Teknologi MARA, Selangor, Malaysia; 6 Department of Internal Medicine, Malacca Hospital, Malacca, Malaysia; 7 Department of Internal Medicine, Taiping Hospital, Perak, Malaysia; 8 Department of Internal Medicine, Selayang Hospital, Selangor, Malaysia; 9 Department of Internal Medicine, Tuanku Jaafar Hospital, Negeri Sembilan, Malaysia; 10 Department of Internal Medicine, Kuala Lumpur Hospital, Kuala Lumpur, Malaysia; 11 Department of Internal Medicine, Tengku Ampuan Rahimah Hospital, Selangor, Malaysia; 12 Department of Family Medicine, Faculty of Medicine and Health Sciences, Universiti Putra Malaysia, Selangor, Malaysia; Fiji National University, FIJI

## Abstract

**Objective:**

To examine the effectiveness and safety of non-pharmacological interventions to reduce bone loss among post-stroke adult patients.

**Data sources:**

Cochrane Central Register of Controlled Trials (CENTRAL), Cochrane Database for Systematic Reviews, MEDLINE, CINAHL, ScienceDirect, Scopus, PubMed and PeDRO databases were searched from inception up to 31st August 2021.

**Methods:**

A systematic review of randomized controlled trials, experimental studies without randomization and prospective cohort studies with concurrent control of non-pharmacological interventions for adult stroke patients compared with placebo or other stroke care. The review outcomes were bone loss, fall and fracture. The Cochrane Risk of Bias Tools were used to assess methodological quality, and Grading of Recommendations, Assessment, Development and Evaluations Framework to assess outcome quality. Synthesis Without Meta-Analysis (SWiM) was used for result synthesis.

**Results:**

Seven studies (n = 453) were included. The methodological and outcome qualities varied from low to moderate. There were statistically significant changes between the intervention and parallel/placebo group in bone mineral density, bone mineral content, cortical thickness and bone turnover markers with specific physical and vibration therapies (p<0.05). Falls were higher in the intervention group, but no fracture was reported.

**Conclusion:**

There was low to moderate evidence that physical and vibration therapies significantly reduced bone loss in post-stroke patients at the expense of a higher falls rate. The sample size was small, and the interventions were highly heterogeneous with different duration, intensities and frequencies. Despite osteoporosis occurring with ageing and accelerated by stroke, there were no studies on vitamin D or protein supplementation to curb the ongoing loss. Effective, high-quality non-pharmacological intervention to improve post-stroke bone health is required.

## Introduction

The risk for hip fracture is quadrupled in stroke survivors compared to healthy individuals [1, 2], attributable mainly to falls and disuse osteoporosis in the paretic side [3–5]. A study by Ramnemark *et al*. [5] showed that fracture risk increased from 4% in the first year after stroke to 15% and 24% after 5 and 10 years, respectively. Among stroke patients with unilateral persisting paresis at the time of fracture, up to two-thirds had their fractures on the paretic side [2]. Bone loss was significant in the paretic limb due to loss of muscle tensile strength and immobility. However, it is also present, albeit at a lesser degree, in the non-paretic limb [6]. A recent study showed that milder stroke and osteoporosis, but not stroke type, to be significantly associated with fracture risk [7]. Despite this evidence since 1957, patients with recent strokes were still not adequately screened for osteoporosis [8].

Stroke patients with osteoporosis were found to have a poorer Modified Rankin Score (MRS 2 or more) at three months than those without [9]. From an inversed U-shaped relationship found between fall and MRS, those with MRS ≥2 were more likely to fall than those with lower or higher functional status. This scenario can be due to the low physical activity in the lower functional status group and intact motor-sensory function in the higher functional status group [10]. Therefore, stroke survivors with underlying osteoporosis were more likely to have MRS 2 or more (poor function) and at increased risk of fall, leading to injuries and fractures. Osteoporosis itself lowers the quality of life, increases disability-adjusted life span, and cause a substantial financial burden to the country’s health system [11].

Bone loss started in the early days post-stroke and progressively worsened until the 3^rd^-4^th^ month before plateauing off afterwards [12, 13]. This loss is probably due to sudden immobility and reduced muscle function, leading to disuse atrophy. Osteoporosis is equally distributed in men, women and younger patients with stroke [14, 15]. It is a concern as many developing countries report strokes occurring in a younger cohort [16]. The medical fraternity has highlighted this risk post-stroke and has recommended assessing fall and fracture risk for all adult stroke patients [17–19]. However, it does not make a firm recommendation on the interventions to prevent the bone loss that occurs post-stroke.

Non-pharmacological management remains the mainstay for bone health until osteoporosis or fragility fractures occur. Exercise benefits in preventing falls and fractures among the general population [20–22]. However, findings may not necessarily translate to the stroke cohort, and it is uncertain whether such non-pharmacological interventions are feasible and effective. A meta-analysis found that no strong recommendation can be made on daily Vitamin D and calcium intake for fracture prevention, due to methodological problems, with unknown efficacy or safety of high dose Vitamin D in high-risk individuals [23]. Therefore, this review aimed to examine the effectiveness and safety of non-pharmacological interventions (physical therapy, nutrition and vitamin supplements) that has been done previously to prevent bone loss among post-stroke individuals.

## Methods

### Search strategy and selection of studies

A systematic review of the literature was carried out. The following online databases were searched for articles published from inception to 31st August 2021: Cochrane Central Register of Controlled Trials (CENTRAL), Cochrane Database for Systematic Reviews, MEDLINE, CINAHL (Cumulative Index to Nursing and Allied Health Literature), ScienceDirect, Scopus, PubMed and PeDRO. The search was initially performed from 24^th^ November 2020 until 1^st^ December 2020, and a search re-run was done from 29^th^ September 2021 till 30^th^ September 2021. We also searched the reference lists of primary studies included in the review.

Search terms were stroke, cerebral infarct, hemiplegia, cerebrovascular accident, osteoporosis, bone resorption, bone density, bone loss, exercise, nutrition, vitamin, multifactorial. The search terms used for each database were described in S1 File. Due to limited translation resources, searches were limited to human studies and the English language. The protocol for this systematic review was registered in PROSPERO (CRD42021231970).

The first reviewer (HMS) downloaded all retrieved studies into a reference manager software (EndNote 20), removed the retracted articles and duplicates. According to the eligibility criteria, the titles and abstracts were screened for full-text review by two reviewers (NAT, LSP). All retrieved full text articles were reviewed by two independent reviewers (LHT, LWC), with the exclusion criteria documented. The discrepancy between the two reviewers was resolved at each stage by a discussion with a third reviewer (TO) and recorded in a table form, with reasons for exclusion stated.

### Eligibility criteria

The review question was built on the PICOS (Participants, Interventions, Comparisons, Outcome and Study Design) framework, as shown in Table 1.

**Table 1:** The development of review questions based on PICOS framework.

Item	Description
Participants	Participants aged 45 years old and above who had sustained a stroke of any type or severity.
Intervention	Non-pharmacological interventions (nutritional intervention, dietary supplementation, exercise, and physical activity).
Comparison	At least one comparator group comprised participants receiving placebo or other stroke care, which was non-pharmacological.
Outcome	Bone loss was assessed by between-group changes in areal bone mineral density, volumetric bone mineral density, bone mineral content and any other surrogate marker of bone quality measured by dual-energy x-ray absorptiometry (DXA), peripheral quantitative CT (pQCT) or peripheral ultrasound; and the changes in bone turnover markers (N-terminal propeptide of type 1 procollagen (P1NP), Osteocalcin (OC), C- and N-terminal telopeptide of type 1 collagen (CTX and NTX)). Falls, fractures, treatment adherence and side effects during the study period (either self-reported, clinical records or diagnosis made by healthcare professionals).
Study Design	Randomized controlled trials (RCTs), experimental studies without randomization and prospective cohort studies with concurrent control.

Any pharmacological interventions including anti-osteoporotic medications, hormonal treatment and traditional, complementary medicine were excluded. Case reports, grey literature, case-control studies, studies with historical controls and cross-sectional studies were also excluded as these were regarded to be of lower evidence in assessing intervention effectiveness.

### Evaluation of methodological quality

Two review authors (HMS, YYH) independently assessed the risk of bias for each study using the criteria outlined in the Cochrane Handbook for Systematic Reviews of Interventions [24]. Randomized trials were evaluated using the ROB-I tool, while non-randomized trials used ROBINS-I. Disagreement between two reviewers was resolved by discussion with a third reviewer (TO). The data were analyzed using RevMan 5.4.

### Effect measures

Synthesis without meta-analysis (SWiM) [25] was carried out because the interventions and outcome measures were too diverse to yield a meaningful summary effect estimate.

### Data extraction and synthesis

Data extraction from included studies was performed independently using a standardized form piloted by two reviewers (NH/RN). Disagreement between two reviewers was resolved by discussion with a third reviewer (TO). Studies were analyzed separately according to their type of interventions. Continuous data were presented as mean differences between groups. Heterogeneity between studies in each type of intervention was tabulated, and included modifiers were gender, age, type and duration of a stroke, baseline bone health, mobility status and settings (recruitment and intervention).

### Certainty of synthesis

All studies had their characteristics and their outcome qualities assessed according to the Grading of Recommendations Assessment, Development and Evaluation (GRADE) system [26] by two reviewers (HMS, YYH). Any discrepancies were resolved by discussion with a third reviewer (SSG). Data were analyzed using the online GRADEpro GDT software. Certainty of evidence assessed using the GRADE framework is shown in S1 Table. We included all selected studies in the synthesis regardless of the methodological or outcome quality because of the few articles available on this particular topic.

## Results

**Fig 1:**
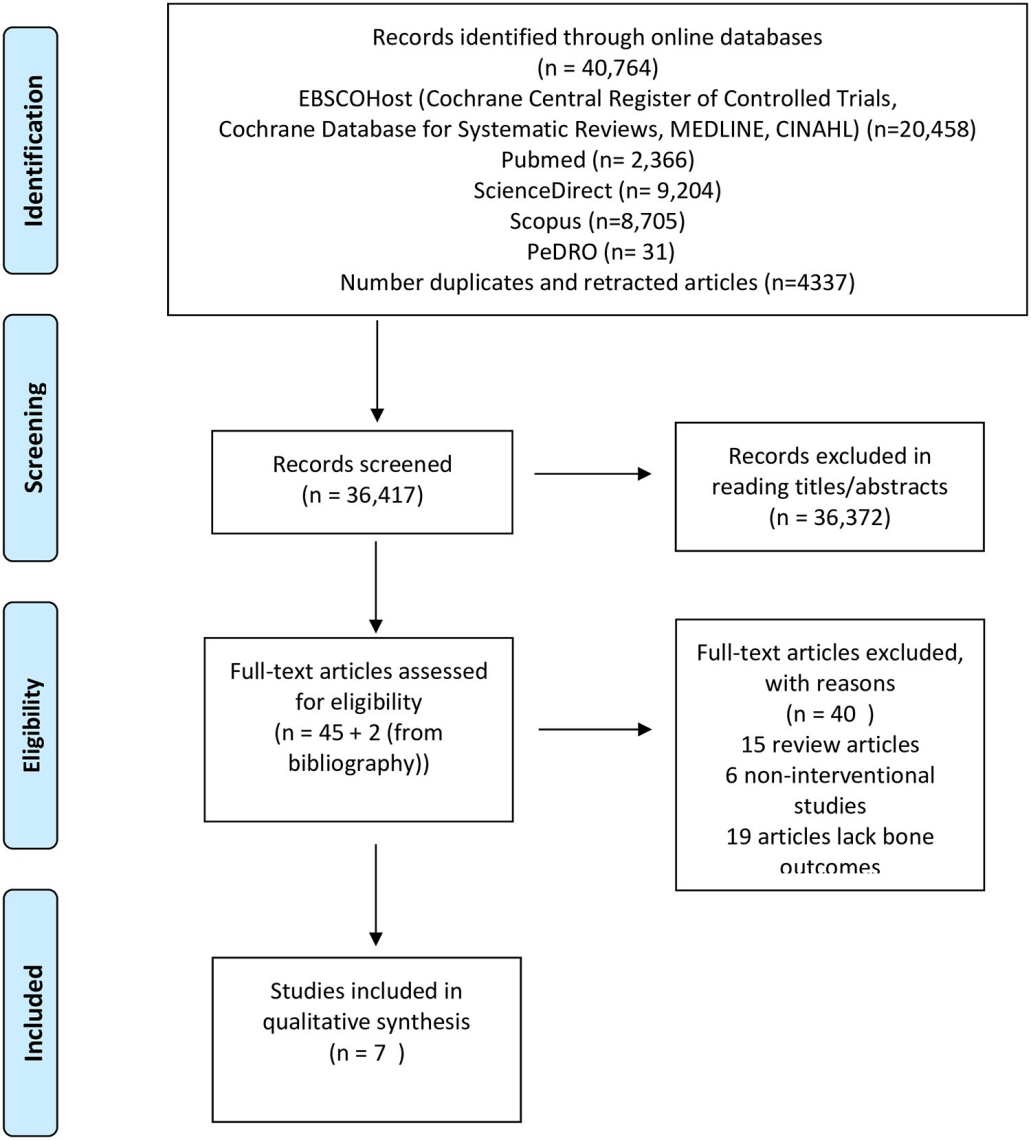
PRISMA flow diagram for study selection.

Our initial online database yielded 40,764 publications, of which seven articles were included in this systematic review. The PRISMA flowchart of the search and selection of studies included in this review are presented in Fig 1.

### Description of included studies

#### Participants

This review included 453 participants from Canada, Japan, Hong Kong and China. Two-thirds of the participants were men (63.6%, n = 288). Recruitment was mainly from the community [27–32] and only one study included hospitalized stroke patients [33]. The mean age of participants in all studies was 62 years (ranging between 46 and 74 years old). The sample size of included trials ranged from 11 to 129 participants. The median sample size was 63 participants in total. The stroke diagnosis to recruitment duration ranged from one week to 9.3 years, with an average of 5 years. Six studies reported the type of stroke [27, 28, 30–33], with 251 out of 432 participants (58.1%) having an ischemic stroke. Five studies reported participants’ mobility upon recruitment and stroke severity [27–30, 32], whereby all participants could walk independently, with or without walking aids. In addition, they were also required to stand for more than 1.5 minutes [30], pedal a stationary cycling ergometer at least 60% heart rate [27] and be able to walk for at least 10 meters [27–29]. Thirty-six out of 63 participants (57%) in two studies respectively have a stroke severity of American Heart Association (AHA) Stroke Functional Class II [27, 28]. One study [29] included those with a moderate to severe impairment in the paretic lower limb, while another study [30] included those with Chedoke McMaster Stroke Assessment (CMSA) paretic leg score 4 out of 7. Three studies [28, 29, 31] reported baseline bone health status of the participants, with 47 out of 95 (49.5%) participants having osteopenia and 20 (21.1%) having osteoporosis. All participants in these studies underwent DXA before the interventions, and no study reported falls or fracture history at baseline. The main characteristics of participants are summarised in Table 2.

**Table 2:** The main characteristic of participants in the included studies.

Authors	Country	Year of age of subjects (Mean age±SD)	Number of participants and Gender	Stroke Characteristic			Participants with osteoporosis/osteopenia (n) and baseline bone health status(mean)
				Time from stroke to recruitment(year)	Type of stroke	Stroke severity and mobility upon recruitment	
Pang, 2006	Canada	I = 63.9±7.0 C = 63.7±7.6	N = 63; I = 32, C = 31 M = 42, F = 21	4% site: I = 6.1 C = 4.7 50%: I = 5.6 C = 5.0	Haemorrhagic = 36	Severity: AHA Stroke Functional Class II (N = 36) Walk >10m independently (with or without walking aids) Pedal a stationary cycling ergometer at least 60% heart rate.	Osteopenia/Osteoporosis = N.A At 4% tibial(mg/cm^3^)(paretic): I = BMD_trab_ 217.1 C = BMD_trab_ 214.7 At 50%(paretic): I = BMD_cort_ 1143 C = BMD_cort_ 1156.3
Pang, 2005	Canada	I = 65.8±9.1, C = 64.7±8.4	N = 63; I = 32, C = 31 M = 37, F = 26	I = 5.2 C = 5.1	Ischemic = 37	Severity: AHA Stroke Functional Class II (N = 36) Walk >10m independently (with or without walking aids)	Paretic side: Osteopenia = 31 Osteoporosis = 9 BMD (g/cm^2^) paretic femoral neck: I = 0.73. C = 0.72
Han, 2017	China	M = 65.34±5.2, F = 68.45±6.54	N = 129; M30 = 25, M60 = 25, M90 = 25, F30 = 18, F60 = 18, F90 = 18 M = 75; F = 54	Acute (hospitalised patients)	Ischemic = 95	N.A	Osteopenia/Osteoporosis = N.A BMD (g/cm^2^) paretic femoral neck: M30 = 0.808 M60 = 0.819 M90 = 0.817 F30 = 0.729 M60 = 0.718 F90 = 0.726
Shimizu, 2002	Japan	I = 62.6±9.2, C = 56.8 ± 16.8	N = 11 I = 5, C = 6 M = 8; F = 3	I = 4.6 C = 4.2	Ischemic = 7, Hemorrhage = 3, SAH = 1	N.A	Osteoporosis = 11 BMD (g/cm^2^) paretic arm: I = 0.36 C = 0.94
Pang, 2010	Hong Kong	I = 64.6±7.2, C = 64.5± 6.2	N = 21; I = 10, C = 11 M = 14; F = 7	I = 7.3 C = 9.3	N.A	Severity: Mod-severe motor impairment in paretic lower limb. Walk ≥10m with or without supervision, with or without walking aids. Tolerate physical activity for about an hour with intermittent rest.	Osteopenia = 16 Osteoporosis = N.A Total hip BMD (g/cm^2^) I = 0.814 C = 0.840
Pang, 2013	Hong Kong	I = 57.3±11.3, C = 57.4±11.1	N = 82; I = 41, C = 41 M = 58; F = 24	I = 4.6 C = 5.3	Ischemic = 41	Severity: CMSA paretic leg score 4 out of 7. Walk independently and able to stand for more than 1.5minutes (with or without aid)	Osteoporosis/Osteopenia = N.A CTX (ng/ml): I = 0.43 C = 0.49 BAP (ng/ml): I = 19.00 C = 22.33
Yang, 2021	Hong Kong	20Hz group = 60.4±5.9 30Hz group = 59.0±7.0	N = 84 20Hz = 42, 30Hz = 42 M = 54, F = 30	20Hz: 4.6±3.7 30Hz: 4.5±3.4	Ischemic = 44 Haemorrhagic = 40	Able to stand for at least 1 min with hand support Fugl-Meyer Lower Limb Score: 24.0±3.5	Osteopenia/Osteoporosis = N.A NTx(nM BCE): 20Hz: 6.1±3.7 30Hz: 6.3±4.2

AHA = American Heart Association; BAP = Bone Alkaline Phosphatase; CMSA = Chedoke McMaster Stroke Assessment; C = Control; CTX = C-terminal telopeptide of type 1 collagen; F = Female; I = Intervention; M = Male; N = Total number; NTx: serum cross-linked N-telopeptides of type I collagen; SAH = Subarachnoid haemorrhage; SD = Standard deviation.

#### Study design and intervention

**Fig 2:**
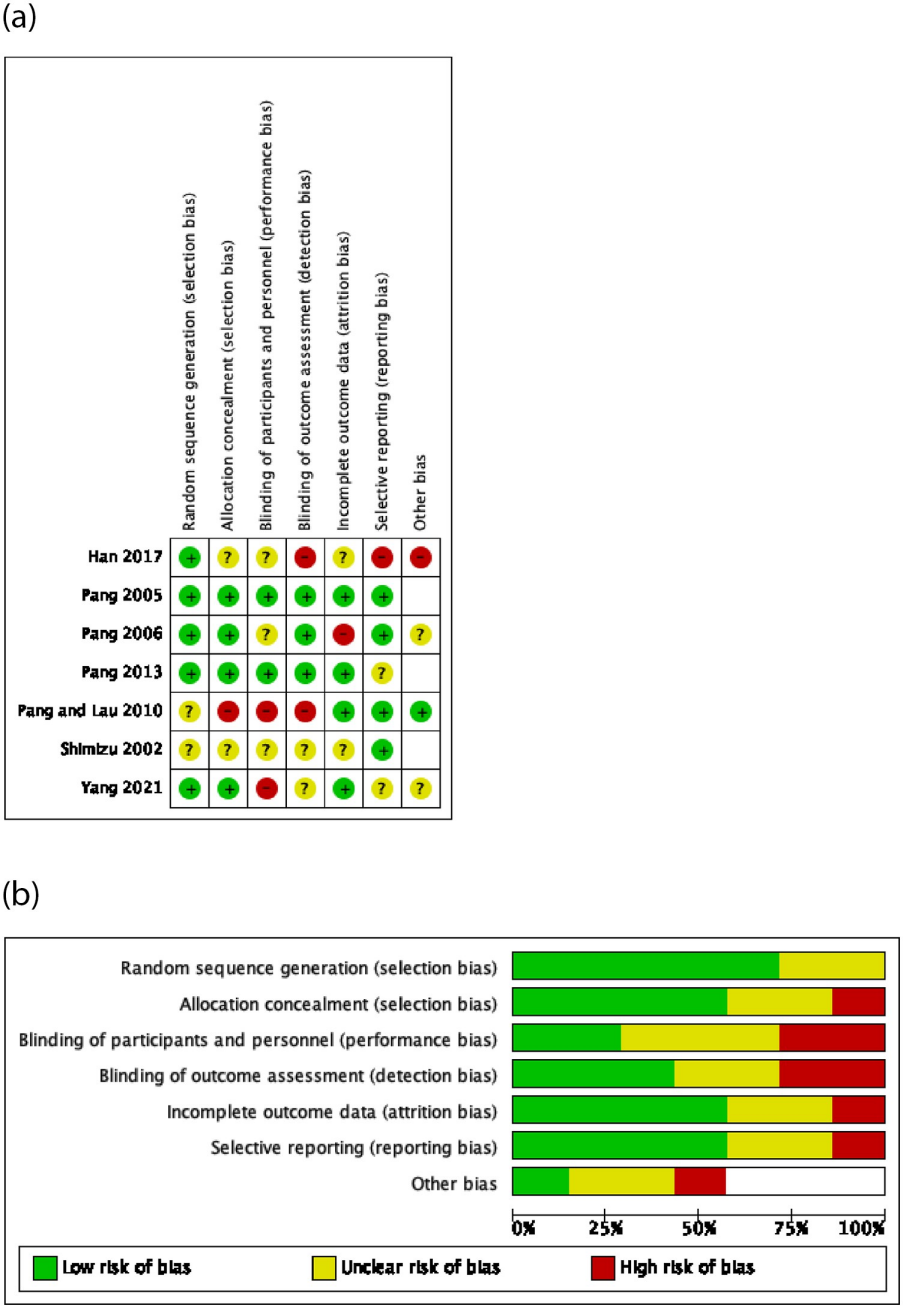
(a). Risk of Bias (ROB) analysis for each study. (b). Summary of risk of bias across the studies.

Included studies were 4 RCTs [27, 28, 30, 31], two randomized, parallel groups without control studies [32, 33] and one quasi-experimental study [29]. The studies were published between 2002–2021. The methodological quality was found to be low to moderate. Fig 2(a) and 2(b) summarise the risk of bias (ROB) for each study and across studies, respectively.

The interventions to prevent post-stroke bone loss were physical and whole-body vibration (WBV) therapies. The studies employed a substantial variety of physical therapy interventions, intensity, frequency, and duration. Resistance exercises in the lower limbs by sit-to-stand, weight-bearing activities and partial squats [27–29, 33] and upper limb by squeezing a ball [31] were employed. Balance exercises by alternate stepping onto low risers, standing on a balance disc, standing up with bodyweight support harness and progressive toe rises [27–29] as well as aerobic exercises using a treadmill, brisk walking and increasing speed of movement were also carried out [27–29]. Two studies used a specific device that emitted vibration frequencies (20-30Hz) [30, 32]. One study [33] had required all study participants to have essential lifestyle modifications such as adequate protein, vitamin D and calcium intake. The study designs and interventions are summarised in Table 3.

**Table 3:** Study designs and interventions employed in the included studies.

Authors	Design	Intervention			Comparator
		Physical Exercise	Nutrition	Others	
Pang, 2006	RCT	Weight-bearing activities and aerobic exercises, static and dynamic balance exercises, functional and muscle strengthening exercises during weight-bearing. Duration:19 weeks Frequency: 1-hour sessions, 3 sessions per week Station 1: Initially, 10 minutes of continuous exercise with increments of 5 minutes weekly up to 30 minutes as tolerated. As tolerated, an increase of 10% HRR per 4 weeks, up to 70%–80% HRR. Station 2: Standing on a balance disc (33 cm in diameter and 6 cm in height) or a tilting board (40cm in length × 37.5 cm in width × 7.5 cm in height), tandem walking, walking in different directions and kicking a ball with either foot. Reducing arm support and increasing speed of movement as tolerated. Station 3: Partial squats and toe raises while holding hand weights. Increasing repetition from 2 sets of 10 to 3 sets of 15 as tolerated.	N.A	N.A	Seated upper extremity exercise program Upper extremity muscle strengthening exercises, passive or self-assisted range of motion exercises, and functional training. For participants with < 20° of active wrist extension, electrical stimulation to the wrist extensor muscles was applied
Pang, 2005	RCT	Resistance, aerobic, mobility and balance exercises. Duration: 19 weeks Frequency: 1 hour-3 sessions per week Station 1: Brisk walking; sit-to-stand: progressed by reducing the height of chair; alternate stepping onto low risers: progressed by increasing the height of the stepper and/or reducing arm support. 10 minutes initially, with increment of 5 minutes weekly, up to 30 minutes continuously as tolerated. Started at 40–50% HRR, with increment of 10% HRR every 4 weeks, up to 70–80% HRR, as tolerated. Station 2: Walking in different directions. Progressed by reducing arm support and/or by increasing speed of movement. Station 3: Partial squats progressed by increasing movement magnitude; toe rises progressed from bilateral to unilateral rises on either side. Increasing number of repetitions (from 2 sets of 10 to 3 sets of 15) and/or by reducing arm support.	N.A	N.A	Station 1: Shoulder muscle strength: Theraband exercises (progressed by increasing the resistance of Theraband and increasing number of repetitions). Station 2: Elbow/ wrist muscle strength and range of motion: dumbbell/wrist cuff weight exercises; passive or self-assisted range of motion to paralyzed joints; upper extremity weight-bearing on physio ball. Station 3: Hand activities: hand muscle strengthening exercises, electrical stimulation to wrist extensors.
Han, 2017	Randomized, parallel group, no control	Daily standing bed weight training Duration:3 months Frequency: 5 days/week, 30min, 60min or 90min. -M30/F30 were required to take weight training for 30 min, once per day. -M60/F60 were required to take weight training twice per day. -M90/F90 were required to take weight training three times per day	All participants were advised on: i) Balanced diet with abundant calcium, protein and low salt; and ii) oral CaCO_3_+D3, one pill 2x/day = 1,200 mg/day calcium and 250 IU/day vitamin D3	All participants were advised on: Outdoor activities and sunshine; No smoking or drinking alcohol; Fall prevention.	Comparison between the 30min, 60min and 90min groups, within male or female. No placebo groups.
Shimizu, 2002	RCT	Home exercises Duration: 1–3 years (mean 1.83 years) Frequency: Daily, 3 times a day, at least 3 times a week. Participants sitting on a chair with elbow flexed and arm resting, were given 3 toy balls with different hardness; soft, regular and hard. Participants were asked to squeeze each ball few times to determine and choose which hardness suitable for them, then slowly squeeze the ball as hard as possible 10 times with the uninvolved hand and then 10 times with the involved hand.	N.A	N.A	Standard physical therapy, not otherwise stated in detail.
Pang, 2010	Quasi-experimental	Aerobic, resistance, balance Duration:6 months Frequency:1-hour for 2 sessions/week, total 52 sessions A harness that connected to body weight support (BWS) was worn by participants. It was set/ reduced to the level that the hemiparetic leg could still support the body weight during the stance phase gait with the aim to withdraw the BWS as soon as the participant was able. Treadmill speed increased by 0.045 m/s (0.1mph) and the duration of walking was gradually increased as tolerated (up to 20 minutes).	N.A	N.A	Usual activities the community e.g. leisure walking, light household task and cleaning household.
Pang, 2013	RCT	Vibration therapy Duration: 8 weeks Frequency: 3x/ week, a total of 24 sessions The exercise used a commercially available device that generated vibration. Six different exercises while standing on the vibration platform with frequency range for the vibration signals was 20-30Hz.	N.A	N.A	The participants performed the same exercises on the same WBV platform but without vibration (i.e. the device was turned off)
Yang, 2021	Randomized, parallel group, no control	Vibration therapy Duration: 8 weeks Frequency: 3x/ week, a total of 24 sessions The exercise used a commercially available device that generated vibration. The vibration was provided in 1-min bouts, with a 1-min rest period between bouts. -20 Hz frequency group: 12 WBV bouts/session -30 Hz frequency group: 8 WBV bouts/session (i.e., 14,400 loading cycles). Total WBV dosage for each session was equivalent between groups. All participants held on the handrail to maintain balance.	N.A	N.A	Comparison between the 20Hz and 30Hz vibration frequency. No placebo groups.

RCT = Randomized, controlled trial; N.A = Not available; WBV = Body vibration

### Intervention effects

Measurement tools and results of the included studies are summarised in Table 4.

**Table 4:** Result of individual studies.

Authors	Bone-related outcome	Tool	Time to measurement	Result (mean difference)		Adverse effects
Pang, 2006 [27]	Effect on lower extremities BMD, BMC and cortical thickness	pQCT	1–2 weeks before intervention program and again within 1–2 weeks after the termination of program.	At 4% (paretic): *BMC_trab:_ I = 5.6±6.7 C = -0.5±10.8 **p = 0.048** BMD_trab_: I = 1.3±1.9 C = 0.1±2.9 p = 0.14 Non-paretic (4% and 50%): No significant difference	At 50% (paretic): BMC_cort_ I = 0.4±2.9 C = -0.4±1.4 p = 0.21 BMD_cort:_ I = -0.2±1.0 C = -0.1±0.7 p = 0.73 *Cortical thickness: I = 0.4±2.2 C = -0.9±1.9 **p = 0.026**	Five falls in intervention group.
Pang, 2005 [28]	Effect on hip BMD	DXA	Immediately before and immediately after the end of the interventions.	*Change in BMD (paretic): I = -0.00 (CI -0.02–0.01) C = -0.02 (CI -0.03 to -0.01) **p = 0.043** No significant difference between group in non-paretic leg.		Hip protectors worn in intervention group. Five falls in intervention the group. One fall in the control group. No injury reported.
Han, 2017 [33]	Effect of training time on BMD	DXA	Before and 3 months after weight training.	Change in BMD between groups: *M60: Lumbar B = 0.819±0.133 A = 0.906±0.137 **p<0.01** Femur (paretic) B = 0.720±0.124 A = 0.785±0.118 **p<0.05**	*F90 Lumbar B = 0.726±0.128 A = 0.805±0.117 **p<0.05** Femur (paretic) B = 0.681±0.122 A = 0.743±0.128 **p<0.05**	N.A.
Shimizu, 2002 [31]	Effect on BMC and BMD	DXA	Before and at the end of the program	No statistical difference of BMD or BMC for the affected arm (n = 4) when compared to the sound arm (n = 4) or control group (n = 3) in the ischemic stroke group. No report on haemorrhagic stroke and p value.		No adverse effects. Easy to perform.
Pang, 2010 [29]	Effect on hip BMD and cortical thickness	DXA pQCT	1 week prior to the intervention, and within 1 week after termination of the program	Changes of BMD total hip (paretic): I = -0.004±0.008 C = -0.005±0.019 p = 0.798	Changes at 66% (paretic): BMD_cort_: I = 5.7±59.1 C = -7.5±76.2 p = 0.481. *Cortical thickness: I = 0.1 ± 0.1 C = -0.0 ± 0.1 **p = 0.018**	No adverse events. Participants satisfied and keen to continue long-term.
Pang, 2013 [30]	Effect on bone turnover marker	CTX BAP	At baseline, immediately after and 1 month after termination of the training.	Post WBV CTX (ng/ml): I = 0.46 (0.29) C = 0.47(0.25), BAP (ng/ml): I = 18.91 (7.00) C = 22.42 (8.28)	Reported no significant effect between group. No p value reported.	No major adverse event.
Yang, 2021 [32]	Effect on bone turnover marker	NTx	At baseline and at the end of the eight-week intervention period.	*Change score (pre-post): 20Hz: −2.2±3.4**, p<0.001** 30 Hz: −2.7±4.0, **p<0.001**	Change scores mean difference between groups: −0.5 (−2.1,1.1), p = 0.540	No adverse event.

A = After; B = Before; BAP = Bone specific alkaline phosphatase; BMD = Bone mineral density(g/cm2); BMC = Bone mineral content; C = Control group; CTX = C-Telopeptide of Type-1 collagen cross-link; DXA = Dual-energy X-ray absorptiometry; I = Intervention group; NTx: serum cross-linked N-telopeptides of type I collagen; pQCT = peripheral quantitative computed tomography; WBV = Whole body vibration.

*Significant result written in bold.

#### Bone mineral density

Two [28, 33] out of 4 studies [28, 29, 31, 33] that measured this outcome using DXA demonstrated a significantly reduced bone loss or improved bone mineral density (BMD) in post-stroke subjects following the specified interventions. An hour of three-times-weekly community-based fitness and mobility exercise program for 19 weeks [28] was able to reduce bone mineral density loss in the intervention group, in the paretic lower limb (-0.00 (CI -0.02–0.01) vs -0.02 (CI -0.03 to -0.01),p = 0.043), but not in the non-paretic limb. It was also shown that standing weight training of a minimum of 60min/day and 90min/day for three months was needed for males and females, respectively, for a statistically significant BMD improvement as measured by DXA in the lumbar spine (p<0.05) and femur (p<0.05) [33]. Treadmill exercises with bodyweight support did not statistically improve BMD in the paretic leg [29]. Home exercise by squeezing a ball did not result in a statistical difference in BMD in the arms of osteoporotic post-stroke patients [31]. Two studies [27, 29] that measured this outcome using pQCT at 4% area (BMD_trab_), 50% area(BMD_cort_) [27] and 66% area(BMD_cort_) [29] of the paretic lower limb did not show any significant difference.

#### Bone mineral content

Two studies reported this outcome [27, 31] using pQCT and DXA, respectively. An hour, three sessions a week of weight-bearing activities and aerobic exercises, static and dynamic balance exercises, as well as functional and muscle strengthening exercises during weight-bearing for 19 weeks, resulted in a significant increase in bone mineral content (BMC) at 4% area of the paretic leg (BMC_trab_,5.6±6.7 vs -0.5±10.8,p<0.05), but not in the non-paretic leg and at 50% area (BMC_cort_,p = 0.21) [27]. No statistical difference in the BMC in the upper limb after a home exercise was found [31].

#### Cortical thickness

Two studies measured this outcome by using pQCT [27, 29]. Weight-bearing activities, aerobic exercises, balance exercises, and strengthening exercises during weight-bearing for 19 weeks [27] led to a significant change in mean difference between the two groups at 50% area of the paretic leg (0.4±2.2 vs -0.9±1.9, p = 0.026). A treadmill exercise with bodyweight support for six months [29] led to a significant change in mean difference between the two groups at 66% area of the paretic leg (0.1 ± 0.1 versus -0.0 ± 0.1,p = 0.018).

#### Bone turnover markers

Two studies reported on bone turnover markers [30, 32]. There was no significant effect of WBV (20-30Hz) administered for eight weeks on C-Telopeptide of Type-1 collagen cross-link (CTX) and Bone-specific Alkaline Phosphatase (BAP) [30]. However, there was a significant reduction of bone loss assessed by serum Cross-linked N-telopeptides of Type I collagen (NTx) in both 20Hz and 30Hz vibration frequency groups after eight weeks (−2.2±3.4 and −2.7±4.0, respectively,p<0.001) [32].

#### Falls

Six studies [27–32] reported adverse incidents. Two studies [27, 28] specifically mentioned fall incidents, while four studies [29–32] mentioned no adverse effects in general, without specifying falls incidents. One study [33] did not report on the adverse incident at all. Among the six studies (n = 324) that assessed adverse effects, there were ten falls in the intervention group (4.9%,n = 204) and one in the control group (0.8%, n = 120). None of the falls resulted in serious injury. The cause of falls was not mentioned.

#### Other adverse events

One study reported other adverse events apart from falls, where two participants in the arm (control) group reported soreness in the shoulder region of the paretic side, which was alleviated after reducing the weight lifted and modifying the exercise [27].

### Certainty of evidence

The quality of evidence regarding bone mineral density, bone mineral content, cortical thickness, bone turnover markers, and falls ranged from low to moderate across the five comparisons in the GRADE Framework, as depicted in S1 Table. This means that we have a low to moderate level of certainty in these results.

## Discussion

From the low to moderate-quality evidence that we gathered, we could not make a firm recommendation on the best approach to prevent bone loss in post-stroke patients. The positive and significant findings of bone loss reduction were found in the bone mineral density, bone mineral content and cortical thickness of the lower extremities among those who underwent upright weight-bearing, balance and aerobic exercises. This finding suggests the potential benefit of physical therapy and exercise in bone loss reduction among post-stroke patients, as previously evidenced among postmenopausal women [22].

The interventions were substantially heterogeneous, mainly included a small number of participants and only one study included acutely hospitalized stroke patients during recruitment [33]. Exercise type, frequency, duration, intensity, and methods were highly heterogeneous. The populations studied also differed widely, from immediate post-stroke to many years post-stroke, with variable functional ability.

Whole-body vibration induced osteogenic effects against age-associated bone mass alteration in older adults via mechanical stimulation [34]. It was shown to reduce bone resorption in postmenopausal women [35, 36]. Among post-stroke patients, vibration therapy at 20-30Hz frequencies reduced bone resorption only when assessed by NTx, but not CTX and BAP [30, 32]. Bone turnover markers could be time-specific and related to nutritional intake before, during and after exercise [37]. For example, CTX should be taken in the early morning in a fasted state, while P1NP is more stable for these changes [38].

An exercise that needs special equipment (for example, a WBV device) may not be feasible to replicate and expand in larger-scale studies without adequate funding [30, 32]. The device usage may be suitable in the research setting. However, from the requirement of having a therapist to ensure an accurate frequency and amplitude used and proper knee flexion of 60^o^ while standing on the vibration platform, it can be cumbersome for home or community setting in the long run [32]. The two studies were carried out using the same vibration device, but patients’ functional status was assessed using different tools at baseline. The study designs were different in which one of them did not have a control group.

The existing studies only mentioned the effects of physical therapies to prevent post-stroke bone loss. Other aspects such as nutrition and vitamin supplements in optimizing bone health were not explored. Nutritional factor plays a role in moderating the bone metabolic response to exercise [37]. Only one study by Han et al [33] employed adequate protein, vitamin D and calcium intake for all its participants. However, monitoring the intake (for example, pill counts and food diary) was not mentioned. The level of serum 25(OH)D was also not monitored in any of the included studies to assess vitamin D deficiency, which is an important aspect of bone health. A study in a developing country [39] showed that a low vitamin D level was prominent among its general population. In the Rotterdam study, Vitamin D deficiency was found to be the consequence of stroke itself [40], a condition found to accelerate bone loss in the proximal femur of post-stroke patients [41]. The risk factors attributed to post-stroke osteoporosis, which are low physical activity and poor nutrition, compounded by multimorbidity and ageing, will increase the risk of falls and fractures. Bone and muscle act as one functional unit, of which muscle strength/mass deterioration after an acute stroke must be intervened early to maintain bone density/strength index [42]. Acute stroke induces muscle hypercatabolism, where protein degradation is higher than protein synthesis. Studies have shown that post-stroke amino acid supplementation could reverse this process. However, the amino acid supplementation effect on bone properties was not measured [43, 44]. Therefore, to have optimum prevention against bone loss, a multi-domain intervention comprising adequate protein, calcium and vitamin D, and individually tailored physical therapy is proposed for acute stroke patients.

### Strength and limitations

This systematic review has identified the evidence so far in improving bone health among post-stroke patients. The comprehensive search strategy employed here would have identified all pertinent published literature on the topic. However, the study had no translation service and was only able to include English language articles. There are limitations with this study as it does not take into consideration the effect of comorbidity, gender, frailty, vitamin D deficiency, and other factors that affect bone loss to determine the effect of these interventions on post-stroke bone health. Postmenopausal women consist 36% of the study population, which means the effect of postmenopausal bone density loss could be underestimated. Another limitation is that most included studies are from the same author, Pang et al, which may influence our findings.

### Implications of the results for practice, policy, and future research

There is a need for further larger-scale studies with cost-effective interventions to be carried out in the future, which may be applicable in developing countries where expert resources and financial support are limited. The intervention may include a combination of aerobic, balance and resistance exercises, with protein and vitamin supplementation that is easy to replicate in multiple centres internationally. Given the complex, multifactorial, and heterogeneous nature of post-stroke bone loss among the global ageing population, interventions simultaneously targeting several risk factors and mechanisms may be required for optimal preventive effects. The risk factors may be shared between the osteoporosis mechanism in general, and loss of muscle strength, balance, and sensory impairment that increase the risk of fall in stroke. Methodological harmonization may be possible, for example, if all participants have a known bone health status at baseline, using validated measuring tools such as DXA and bone turnover markers as well as Modified Rankin Score (MRS) as the basis of functional status. Future studies may include the impact of an intervention on the quality of life as this is an essential aspect of ageing research. Finally, health economic studies alongside clinical trials should be carried out in the future to determine the cost-effectiveness of any intervention for stakeholders’ interest in the long-term implementation of a programme.

## Conclusion

There is low to moderate evidence that specific physical and vibration therapies significantly reduce bone loss in post-stroke patients at the expense of a higher falls rate. However, the small sample size and lack of sub-analysis on comorbidity, gender (except Han et al), frailty, vitamin D deficiency, and other factors that may affect bone loss on the intervention outcomes may limit its applicability and generalizability. An extensive search found no published evidence on the effects of vitamins or protein in preventing post-stroke bone loss. In addition, the interventions were highly heterogeneous. With the global population ageing, a more well-designed randomized controlled trial that involves early post-stroke multi-domain intervention may further establish the value of non-pharmacological intervention in reducing post-stroke bone loss.

## Supporting information

S1 File This is the S1 File on search terms used for each online database. (PDF)

S1 Table This is the S1 Table on summary of certainty of evidence using GRADE system. (TIF)

S1 Checklist PRISMA 2020 checklist. (PDF)
