# Supplementary material for: Non-pharmacological interventions for bone health after stroke: A systematic review
Source: PLoS One. 2022 Feb 23;17(2):e0263935. doi: 10.1371/journal.pone.0263935 (PMC8865685; doi:10.1371/journal.pone.0263935)
Supplement: S1 File — (PDF) [file pone.0263935.s001.pdf]

Search terms used

1. stroke OR poststroke OR post-stroke OR "cerebral infarct" OR "cerebral infarction" OR "brain injury" OR hemiplegia OR haemorrhage OR haemorrhagic OR hemiplegic OR hemiplegia OR hemiparesis OR hemiparetic OR paresis OR paretic OR "Cerebrovascular accident"
2. Osteoporosis OR Fracture OR "bone density" OR "bone mineral density" OR "bone resorption" OR "bone loss" OR densitometry OR "bone turnover" OR "bone mass" OR "bone strength" OR "bone volume" OR "bone area"
3. Multi-factorial OR Multifactorial OR multidisciplinary OR multi-disciplinary OR Multi-domain OR Multidomain OR Combined OR Multimodal OR multi-modal OR Exercise OR Rehabilitation OR Mobilization OR Mobilisation OR training OR therapy OR physiotherapy OR Nutrition OR Diet OR Food OR Consumption OR Protein OR Folate OR Calcium OR "1 alpha-hydroxyvitamin D3" OR Cholecalciferol OR Vitamin OR Carbohydrate OR fibre OR fiber

A) EBSCOHost (Medline, CINAHL, Cochrane Database of systematic review, Cochrane Central Register for Controlled Trials)
   All Fields: (1) AND (2) AND (3)

B) Pubmed
   All Fields: (1) AND (2) AND (3)

C) PEDRo
   (Adv) Stroke, Bone

D) Science Direct
   Terms used (max 8): ((stroke OR hemiplegia) AND (osteoporosis OR bone) AND (nutrition OR vitamin OR training OR exercise))

E) Scopus
   (Title-Abs-Key): (1) AND (2) AND (3)
