# Supplementary figures and images for: Non-pharmacological interventions for bone health after stroke: A systematic review
Source: PLoS One. 2022 Feb 23;17(2):e0263935. doi: 10.1371/journal.pone.0263935 (PMC8865685; doi:10.1371/journal.pone.0263935)

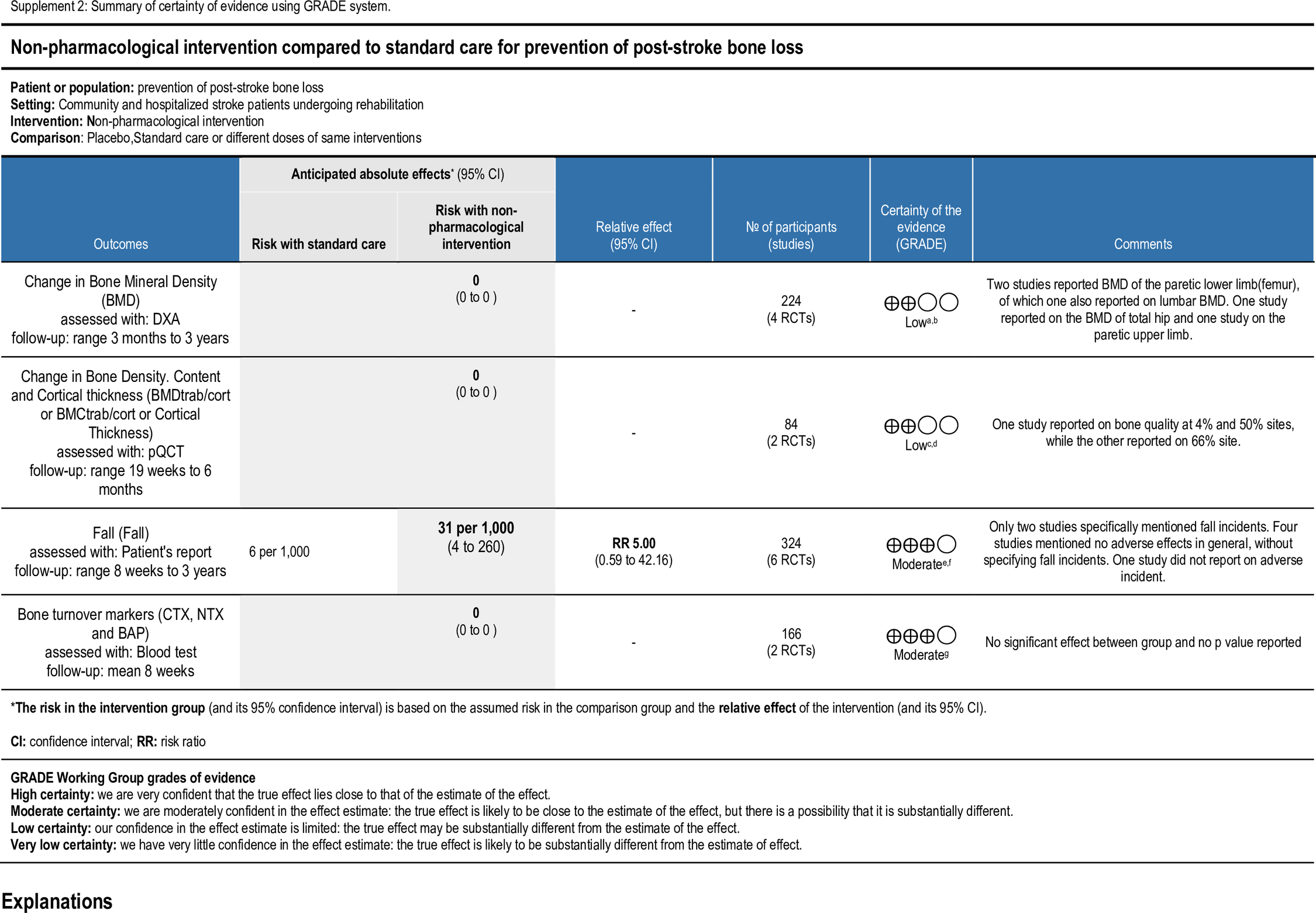

Supplement: S1 Table — (TIF) [file pone.0263935.s002.tif]
